# 2-{2-[(2,6-Dichloro­phen­yl)amino]­phen­yl}ethanol

**DOI:** 10.1107/S1600536810054590

**Published:** 2011-01-08

**Authors:** Nasirullah, Nazar Ul Islam, M. Nawaz Tahir, Ikhtiar Khan

**Affiliations:** a Institute of Chemical Sciences, University of Peshawar, Peshawar, Pakistan; b Department of Physics, University of Sargodha, Sargodha, Pakistan

## Abstract

In the title compound, C_14_H_13_Cl_2_NO, the 2,6-dichloro­anilino unit is roughly planar (r.m.s. deviation = 0.0298 Å) and makes a dihedral angle of 67.71 (4)° with the benzene ring containing the ethanol group. The C–C–O fragment is oriented at a dihedral angle of 64.94 (9)° with respect to its parent benzene ring. The molecular conformation is stabilised by a bifurcated N—H⋯(O,Cl) hydrogen bond. C—H⋯π, O—H⋯π and π–π inter­actions [centroid–centroid distance = 3.5706 (11) Å] stabilize the crystal structure.

## Related literature

For related structures, see: Nasirullah *et al.* (2010 ▶); Rodriguez *et al.* (2007 ▶); Damas *et al.* (1997 ▶); Nawaz *et al.* (2007 ▶); For graph-set notation, see: Bernstein *et al.* (1995 ▶).
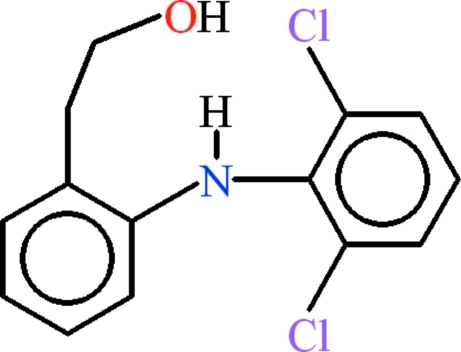


## Experimental

### 

#### Crystal data

C_14_H_13_Cl_2_NO
*M* *_r_* = 282.15
Monoclinic, 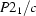

*a* = 8.3521 (3) Å
*b* = 15.0986 (5) Å
*c* = 10.9225 (5) Å
β = 107.180 (1)°
*V* = 1315.93 (9) Å^3^
*Z* = 4
Mo *K*α radiation
μ = 0.48 mm^−1^
*T* = 296 K
0.28 × 0.18 × 0.14 mm

#### Data collection

Bruker Kappa APEXII CCD diffractometer
Absorption correction: multi-scan (*SADABS*; Bruker, 2005 ▶) *T*_min_ = 0.903, *T*_max_ = 0.934
9878 measured reflections
2348 independent reflections
2029 reflections with *I* > 2σ(*I*)
*R*_int_ = 0.022

#### Refinement

*R*[*F* ^2^ > 2σ(*F* ^2^)] = 0.031
*wR*(*F* ^2^) = 0.082
*S* = 1.06
2348 reflections
167 parameters
All H-atom parameters refined
Δρ_max_ = 0.30 e Å^−3^
Δρ_min_ = −0.30 e Å^−3^

### 

Data collection: *APEX2* (Bruker, 2009 ▶); cell refinement: *SAINT* (Bruker, 2009 ▶); data reduction: *SAINT*; program(s) used to solve structure: *SHELXS97* (Sheldrick, 2008 ▶); program(s) used to refine structure: *SHELXL97* (Sheldrick, 2008 ▶); molecular graphics: *ORTEP-3 for Windows* (Farrugia, 1997 ▶) and *PLATON* (Spek, 2009 ▶); software used to prepare material for publication: *WinGX* (Farrugia, 1999 ▶) and *PLATON*.

## Supplementary Material

Crystal structure: contains datablocks global, I. DOI: 10.1107/S1600536810054590/bq2270sup1.cif

Structure factors: contains datablocks I. DOI: 10.1107/S1600536810054590/bq2270Isup2.hkl

Additional supplementary materials:  crystallographic information; 3D view; checkCIF report

## Figures and Tables

**Table 1:** Hydrogen-bond geometry (Å, °) *Cg*1 is the centroid of the C7–C12 ring.

*D*—H⋯*A*	*D*—H	H⋯*A*	*D*⋯*A*	*D*—H⋯*A*
N1—H1⋯Cl1	0.796 (18)	2.628 (18)	2.9888 (16)	109.5 (15)
N1—H1⋯O1	0.796 (18)	2.155 (18)	2.877 (2)	151.0 (18)
O1—H1*A*⋯*Cg*1^i^	0.82	2.58	3.3465 (19)	156
C3—H3⋯*Cg*1^ii^	0.93	2.95	3.791 (2)	152

Symmetry codes: (i) 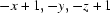; (ii) 
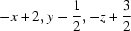
.

**Fig. 1.**
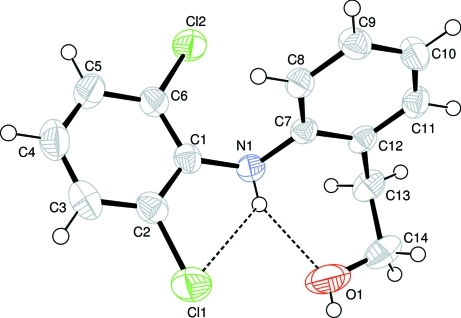
View of (I) with the atom numbering scheme. Displacement ellipsoids are drawn at the 50% probability level. The dotted lines represent the intramolecular H-bonding.

## supplementary crystallographic information

### Comment

The title compound (I, Fig. 1) has been prepared for further derivatization and in continuation to the reduction of carboxylic moieties of some important drugs (Nasirullah *et al.*, 2010) without affecting the medicinally important functional groups.

The crystal structures of (II) *i.e.*, (*Z*)-3-(4-(2-hydroxyethyl)phenylamino)-1-phenylbut-2-en-1-one (Rodriguez *et al.*, 2007) and (III) 2-[2-(hydroxyethyl)phenoxy]benzoic acid (Damas *et al.*, 1997) have been published previously which seems relavant to the present structure.

In (I), the 2,6-dichloroanilinic moiety A (N1/C1—C6/CL1/CL2) and the benzene ring of 2-phenylethanol B (C7—C12) are planar with r. m. s. deviations of 0.0298 Å and 0.0031 Å, respectively. The ethanol moiety C (C13/C14/O1) is of course planar. The dihedral angles between A/B, A/C and B/C are 67.71 (4)°, 10.40 (20)° and 64.94 (9)°, respectively. The molecules are stabilized as monomers due to intramolecular H-bondings of N—H···Cl and N—H···O types (Table 1, Fig. 1) with S(5) and S(7) ring motifs (Bernstein *et al.*, 1995). In the stabilization of molecules C—H···π and  π–π interaction at a distance of 3.5706 (11) Å between the centroids of chloro containing benzene rings play an important role.

### Experimental

A solution of dichlofenac sodium (6.75 mmol) in THF (10 ml) was slowly added to suspension of NaBH_4_ (10 mmol) in THF (10 ml), at room temperature. The mixture was stirred until evolution of hydrogen ceased. Iodine (6.75 mmol) in THF (10 ml) was drop wise added to this mixture. When the addition of iodine was complete, the reaction mixture was refluxed for 8 h and cooled to room temperature. 10 ml of 2 N HCl was added and the mixture was extracted with ethyl acetate. The ethyl acetate layer was washed with 20 ml of 2 N NaOH and then with brine. Finally the ethyl acetate layer was dried over MgSO_4_. On evaporation of the solvent 1.3 g of the crude product was obtained. The product was further purified by column chromatography on silica gel. Light yellow needles of (I) were obtained by recrystallization from ethyl acetate and n-hexane. m.p. of pure product:383 K.

### Refinement

The coordinates of amide H-atoms were refined. Other H-atoms were positioned geometrically (O—H = 0.82, C–H = 0.93–0.97 Å) and refined as riding with *U*_iso_(H) = x*U*_eq_(C, N, O), where x = 1.2 for all H-atoms.

### Crystal data

**Table:**

C_14_H_13_Cl_2_NO	*F*(000) = 584
*M**_r_* = 282.15	*D*_x_ = 1.424 Mg m^−^^3^
Monoclinic, *P*2_1_/*c*	Mo *K*α radiation, λ = 0.71073 Å
Hall symbol: -P 2ybc	Cell parameters from 2029 reflections
*a* = 8.3521 (3) Å	θ = 2.4–25.3°
*b* = 15.0986 (5) Å	µ = 0.48 mm^−^^1^
*c* = 10.9225 (5) Å	*T* = 296 K
β = 107.180 (1)°	Needle, light yellow
*V* = 1315.93 (9) Å^3^	0.28 × 0.18 × 0.14 mm
*Z* = 4	

### Data collection

**Table:**

Bruker Kappa APEXII CCD diffractometer	2348 independent reflections
Radiation source: fine-focus sealed tube	2029 reflections with *I* > 2σ(*I*)
graphite	*R*_int_ = 0.022
Detector resolution: 8.10 pixels mm^-1^	θ_max_ = 25.3°, θ_min_ = 2.4°
ω scans	*h* = −10→9
Absorption correction: multi-scan (*SADABS*; Bruker, 2005)	*k* = −18→18
*T*_min_ = 0.903, *T*_max_ = 0.934	*l* = −13→11
9878 measured reflections	

### Refinement

**Table:**

Refinement on *F*^2^	Primary atom site location: structure-invariant direct methods
Least-squares matrix: full	Secondary atom site location: difference Fourier map
*R*[*F*^2^ > 2σ(*F*^2^)] = 0.031	Hydrogen site location: inferred from neighbouring sites
*wR*(*F*^2^) = 0.082	All H-atom parameters refined
*S* = 1.06	*w* = 1/[σ^2^(*F*_o_^2^) + (0.0334*P*)^2^ + 0.510*P*] where *P* = (*F*_o_^2^ + 2*F*_c_^2^)/3
2348 reflections	(Δ/σ)_max_ < 0.001
167 parameters	Δρ_max_ = 0.30 e Å^−^^3^
0 restraints	Δρ_min_ = −0.30 e Å^−^^3^

### Special details

**Table:**

Geometry. Bond distances, angles *etc*. have been calculated using the rounded fractional coordinates. All su's are estimated from the variances of the (full) variance-covariance matrix. The cell e.s.d.'s are taken into account in the estimation of distances, angles and torsion angles
Refinement. Refinement of *F*^2^ against ALL reflections. The weighted *R*-factor *wR* and goodness of fit *S* are based on *F*^2^, conventional *R*-factors *R* are based on *F*, with *F* set to zero for negative *F*^2^. The threshold expression of *F*^2^ > σ(*F*^2^) is used only for calculating *R*-factors(gt) *etc*. and is not relevant to the choice of reflections for refinement. *R*-factors based on *F*^2^ are statistically about twice as large as those based on *F*, and *R*- factors based on ALL data will be even larger.

### Fractional atomic coordinates and isotropic or equivalent isotropic displacement parameters (Å^2^)

**Table:**

	*x*	*y*	*z*	*U*_iso_*/*U*_eq_	
Cl1	0.75713 (7)	−0.13208 (3)	0.78183 (5)	0.0643 (2)	
Cl2	1.11847 (6)	0.17019 (3)	0.84323 (5)	0.0563 (2)	
O1	0.45224 (19)	0.01954 (11)	0.67674 (19)	0.0809 (6)	
N1	0.80367 (17)	0.06112 (10)	0.74340 (13)	0.0391 (4)	
C1	0.94805 (19)	0.01500 (11)	0.81022 (15)	0.0363 (5)	
C2	0.9460 (2)	−0.07553 (11)	0.83623 (16)	0.0426 (5)	
C3	1.0862 (3)	−0.12212 (13)	0.90315 (18)	0.0533 (7)	
C4	1.2364 (3)	−0.07878 (14)	0.9456 (2)	0.0586 (7)	
C5	1.2460 (2)	0.01071 (14)	0.92471 (19)	0.0540 (7)	
C6	1.1029 (2)	0.05691 (11)	0.85975 (16)	0.0415 (5)	
C7	0.78660 (18)	0.10202 (10)	0.62459 (14)	0.0322 (4)	
C8	0.90748 (19)	0.09185 (10)	0.56114 (16)	0.0373 (5)	
C9	0.8907 (2)	0.13202 (11)	0.44481 (17)	0.0438 (6)	
C10	0.7518 (2)	0.18261 (12)	0.38800 (17)	0.0476 (6)	
C11	0.6306 (2)	0.19269 (11)	0.44974 (17)	0.0428 (5)	
C12	0.64417 (18)	0.15399 (10)	0.56786 (16)	0.0353 (5)	
C13	0.5069 (2)	0.16759 (12)	0.62982 (19)	0.0469 (6)	
C14	0.3779 (2)	0.09453 (15)	0.6051 (2)	0.0599 (7)	
H1	0.719 (2)	0.0399 (12)	0.7492 (18)	0.0469*	
H1A	0.39115	−0.02359	0.65344	0.0971*	
H3	1.07894	−0.18223	0.91928	0.0639*	
H4	1.33248	−0.10997	0.98888	0.0703*	
H5	1.34815	0.04001	0.95408	0.0648*	
H8	1.00146	0.05723	0.59803	0.0448*	
H9	0.97366	0.12489	0.40455	0.0525*	
H10	0.73968	0.20961	0.30925	0.0571*	
H11	0.53635	0.22662	0.41086	0.0514*	
H13A	0.45039	0.22296	0.59905	0.0562*	
H13B	0.55757	0.17322	0.72162	0.0562*	
H14A	0.28150	0.11344	0.63079	0.0719*	
H14B	0.34044	0.08021	0.51453	0.0719*	

### Atomic displacement parameters (Å^2^)

**Table:**

	*U*^11^	*U*^22^	*U*^33^	*U*^12^	*U*^13^	*U*^23^
Cl1	0.0701 (3)	0.0482 (3)	0.0678 (3)	−0.0170 (2)	0.0101 (3)	−0.0019 (2)
Cl2	0.0524 (3)	0.0452 (3)	0.0661 (3)	−0.0084 (2)	0.0096 (2)	0.0026 (2)
O1	0.0531 (9)	0.0673 (10)	0.1199 (14)	−0.0221 (7)	0.0219 (9)	0.0031 (10)
N1	0.0316 (7)	0.0469 (8)	0.0403 (8)	0.0002 (6)	0.0131 (6)	0.0060 (6)
C1	0.0394 (9)	0.0411 (9)	0.0301 (8)	0.0027 (7)	0.0130 (7)	0.0022 (7)
C2	0.0513 (10)	0.0418 (9)	0.0355 (9)	−0.0022 (8)	0.0142 (7)	0.0006 (7)
C3	0.0689 (13)	0.0453 (10)	0.0473 (11)	0.0117 (9)	0.0197 (9)	0.0107 (8)
C4	0.0536 (12)	0.0628 (13)	0.0569 (12)	0.0191 (10)	0.0126 (9)	0.0153 (10)
C5	0.0389 (10)	0.0667 (13)	0.0536 (11)	0.0038 (9)	0.0093 (8)	0.0069 (9)
C6	0.0417 (9)	0.0442 (9)	0.0392 (9)	0.0014 (7)	0.0127 (7)	0.0045 (7)
C7	0.0313 (8)	0.0298 (7)	0.0341 (8)	−0.0033 (6)	0.0077 (6)	−0.0027 (6)
C8	0.0339 (8)	0.0381 (8)	0.0400 (9)	0.0042 (7)	0.0111 (7)	0.0026 (7)
C9	0.0457 (10)	0.0464 (10)	0.0428 (10)	0.0006 (7)	0.0187 (8)	0.0036 (8)
C10	0.0531 (10)	0.0468 (10)	0.0400 (10)	0.0006 (8)	0.0094 (8)	0.0096 (8)
C11	0.0373 (9)	0.0352 (8)	0.0480 (10)	0.0024 (7)	0.0002 (7)	0.0022 (7)
C12	0.0301 (8)	0.0308 (8)	0.0425 (9)	−0.0019 (6)	0.0067 (7)	−0.0071 (7)
C13	0.0359 (9)	0.0479 (10)	0.0569 (11)	0.0038 (7)	0.0139 (8)	−0.0110 (8)
C14	0.0332 (9)	0.0786 (14)	0.0688 (13)	−0.0077 (9)	0.0163 (9)	−0.0147 (11)

### Geometric parameters (Å, °)

**Table:**

Cl1—C2	1.7369 (18)	C9—C10	1.374 (2)
Cl2—C6	1.7288 (17)	C10—C11	1.381 (2)
O1—C14	1.412 (3)	C11—C12	1.390 (2)
O1—H1A	0.8200	C12—C13	1.508 (2)
N1—C1	1.397 (2)	C13—C14	1.510 (3)
N1—C7	1.406 (2)	C3—H3	0.9300
N1—H1	0.796 (18)	C4—H4	0.9300
C1—C2	1.397 (2)	C5—H5	0.9300
C1—C6	1.397 (2)	C8—H8	0.9300
C2—C3	1.377 (3)	C9—H9	0.9300
C3—C4	1.369 (3)	C10—H10	0.9300
C4—C5	1.377 (3)	C11—H11	0.9300
C5—C6	1.385 (3)	C13—H13A	0.9700
C7—C8	1.392 (2)	C13—H13B	0.9700
C7—C12	1.407 (2)	C14—H14A	0.9700
C8—C9	1.377 (2)	C14—H14B	0.9700
			
C14—O1—H1A	109.00	C12—C13—C14	114.67 (15)
C1—N1—C7	122.53 (14)	O1—C14—C13	108.25 (16)
C1—N1—H1	114.1 (13)	C2—C3—H3	120.00
C7—N1—H1	113.6 (14)	C4—C3—H3	120.00
N1—C1—C2	122.23 (15)	C3—C4—H4	120.00
N1—C1—C6	122.27 (15)	C5—C4—H4	120.00
C2—C1—C6	115.46 (15)	C4—C5—H5	120.00
C1—C2—C3	123.25 (17)	C6—C5—H5	120.00
Cl1—C2—C1	118.41 (13)	C7—C8—H8	119.00
Cl1—C2—C3	118.34 (14)	C9—C8—H8	119.00
C2—C3—C4	119.10 (18)	C8—C9—H9	120.00
C3—C4—C5	120.4 (2)	C10—C9—H9	120.00
C4—C5—C6	119.71 (18)	C9—C10—H10	121.00
C1—C6—C5	122.03 (16)	C11—C10—H10	121.00
Cl2—C6—C1	119.81 (13)	C10—C11—H11	119.00
Cl2—C6—C5	118.15 (14)	C12—C11—H11	119.00
N1—C7—C12	119.57 (14)	C12—C13—H13A	109.00
N1—C7—C8	121.37 (14)	C12—C13—H13B	109.00
C8—C7—C12	119.06 (14)	C14—C13—H13A	109.00
C7—C8—C9	121.32 (15)	C14—C13—H13B	109.00
C8—C9—C10	120.24 (16)	H13A—C13—H13B	108.00
C9—C10—C11	118.92 (16)	O1—C14—H14A	110.00
C10—C11—C12	122.44 (16)	O1—C14—H14B	110.00
C7—C12—C13	122.31 (15)	C13—C14—H14A	110.00
C7—C12—C11	118.02 (15)	C13—C14—H14B	110.00
C11—C12—C13	119.67 (15)	H14A—C14—H14B	108.00
			
C7—N1—C1—C2	−117.26 (18)	C4—C5—C6—Cl2	176.57 (16)
C7—N1—C1—C6	65.2 (2)	C4—C5—C6—C1	−2.2 (3)
C1—N1—C7—C8	6.0 (2)	N1—C7—C8—C9	−179.93 (15)
C1—N1—C7—C12	−174.38 (15)	C12—C7—C8—C9	0.4 (2)
N1—C1—C2—Cl1	0.6 (2)	N1—C7—C12—C11	−179.35 (15)
N1—C1—C2—C3	−179.22 (17)	N1—C7—C12—C13	−0.2 (2)
C6—C1—C2—Cl1	178.28 (12)	C8—C7—C12—C11	0.3 (2)
C6—C1—C2—C3	−1.5 (3)	C8—C7—C12—C13	179.44 (15)
N1—C1—C6—Cl2	2.0 (2)	C7—C8—C9—C10	−0.8 (3)
N1—C1—C6—C5	−179.29 (16)	C8—C9—C10—C11	0.3 (3)
C2—C1—C6—Cl2	−175.70 (13)	C9—C10—C11—C12	0.4 (3)
C2—C1—C6—C5	3.0 (2)	C10—C11—C12—C7	−0.7 (2)
Cl1—C2—C3—C4	179.35 (16)	C10—C11—C12—C13	−179.89 (16)
C1—C2—C3—C4	−0.8 (3)	C7—C12—C13—C14	−83.6 (2)
C2—C3—C4—C5	1.8 (3)	C11—C12—C13—C14	95.6 (2)
C3—C4—C5—C6	−0.4 (3)	C12—C13—C14—O1	72.5 (2)

### Hydrogen-bond geometry (Å, °)

**Table:**

Cg1 is the centroid of the C7–C12 ring.

**Table:**

*D*—H···*A*	*D*—H	H···*A*	*D*···*A*	*D*—H···*A*
N1—H1···Cl1	0.796 (18)	2.628 (18)	2.9888 (16)	109.5 (15)
N1—H1···O1	0.796 (18)	2.155 (18)	2.877 (2)	151.0 (18)
O1—H1A···Cg1^i^	0.82	2.58	3.3465 (19)	156
C3—H3···Cg1^ii^	0.93	2.95	3.791 (2)	152

Symmetry codes: (i) −*x*+1, −*y*, −*z*+1; (ii) −*x*+2, *y*−1/2, −*z*+3/2.
